# Role of Phenolic Compounds in Human Disease: Current Knowledge and Future Prospects

**DOI:** 10.3390/molecules27010233

**Published:** 2021-12-30

**Authors:** Md. Mominur Rahman, Md. Saidur Rahaman, Md. Rezaul Islam, Firoza Rahman, Faria Mannan Mithi, Taha Alqahtani, Mohannad A. Almikhlafi, Samia Qasem Alghamdi, Abdullah S Alruwaili, Md. Sohel Hossain, Muniruddin Ahmed, Rajib Das, Talha Bin Emran, Md. Sahab Uddin

**Affiliations:** 1 Department of Pharmacy, Faculty of Allied Health Sciences, Daffodil International University, Dhaka 1207, Bangladesh; mominur.ph@gmail.com (M.M.R.); mdsaidur569@gmail.com (M.S.R.); md.rezaulislam100ds@gmail.com (M.R.I.); 940sumaiya@gmail.com (F.R.); mithilafaria43@gmail.com (F.M.M.); sohelhossain1173@gmail.com (M.S.H.); drmuniruddin@gmail.com (M.A.); 2 Department of Pharmacology, College of Pharmacy, King Khalid University, Abha 62529, Saudi Arabia; ttaha@kku.edu.sa; 3 Department of Pharmacology and Toxicology, Taibah University, Madinah 41477, Saudi Arabia; mmikhlafi@taibahu.edu.sa; 4 Department of Biology, Faculty of Science, Al-Baha University, Albaha 65527, Saudi Arabia; sqassim@bu.edu.sa; 5 Department of Clinical Laboratory, College of Applied Medical Science, Northern Border University, P.O. Box 1321, Arar 9280, Saudi Arabia; Dr.d-d@hotmail.com; 6 Department of Pharmacy, Faculty of Pharmacy, University of Dhaka, Dhaka 1000, Bangladesh; rajibjony97@gmail.com; 7 Department of Pharmacy, BGC Trust University Bangladesh, Chittagong 4381, Bangladesh; 8 Department of Pharmacy, Southeast University, Dhaka 1213, Bangladesh; 9 Pharmakon Neuroscience Research Network, Dhaka 1207, Bangladesh

**Keywords:** phenolic compounds, inflammation, diabetic mellitus, hypertension, cardiovascular disease, antioxidant

## Abstract

Inflammation is a natural protective mechanism that occurs when the body’s tissue homeostatic mechanisms are disrupted by biotic, physical, or chemical agents. The immune response generates pro-inflammatory mediators, but excessive output, such as chronic inflammation, contributes to many persistent diseases. Some phenolic compounds work in tandem with nonsteroidal anti-inflammatory drugs (NSAIDs) to inhibit pro-inflammatory mediators’ activity or gene expression, including cyclooxygenase (COX). Various phenolic compounds can also act on transcription factors, such as nuclear factor-κB (NF-κB) or nuclear factor-erythroid factor 2-related factor 2 (Nrf-2), to up-or downregulate elements within the antioxidant response pathways. Phenolic compounds can inhibit enzymes associated with the development of human diseases and have been used to treat various common human ailments, including hypertension, metabolic problems, incendiary infections, and neurodegenerative diseases. The inhibition of the angiotensin-converting enzyme (ACE) by phenolic compounds has been used to treat hypertension. The inhibition of carbohydrate hydrolyzing enzyme represents a type 2 diabetes mellitus therapy, and cholinesterase inhibition has been applied to treat Alzheimer’s disease (AD). Phenolic compounds have also demonstrated anti-inflammatory properties to treat skin diseases, rheumatoid arthritis, and inflammatory bowel disease. Plant extracts and phenolic compounds exert protective effects against oxidative stress and inflammation caused by airborne particulate matter, in addition to a range of anti-inflammatory, anticancer, anti-aging, antibacterial, and antiviral activities. Dietary polyphenols have been used to prevent and treat allergy-related diseases. The chemical and biological contributions of phenolic compounds to cardiovascular disease have also been described. This review summarizes the recent progress delineating the multifunctional roles of phenolic compounds, including their anti-inflammatory properties and the molecular pathways through which they exert anti-inflammatory effects on metabolic disorders. This study also discusses current issues and potential prospects for the therapeutic application of phenolic compounds to various human diseases.

## 1. Introduction

Polyphenols are secondary plant metabolites that play a vital role in protecting plants from UV radiation and disease attacks [1]. They are naturally occurring compounds present in many foods, including fruits, vegetables, cereals, and beverages. Polyphenols can be found in up to 200–300 mg per 100 g of fresh weight in grapes, apples, pear, cherries, and berries. Their presence is also high in the products manufactured from these fruits. About 100 mg polyphenols are found in a glass of red wine, a cup of tea, or coffee. Polyphenols can also be found in cereals, dried legumes, and chocolate [2,3]. There has been a lot of focus on the potential health benefits of dietary plant polyphenols as antioxidants in the last decade. According to epidemiological research and related meta-analysis, long-term consumption of diets high in plant polyphenols protects against cancer, cardiovascular disease, diabetes, osteoporosis, and neurological diseases [4,5]. Bitterness, astringency, color, flavor, odor, and oxidative stability are all things that polyphenols can help with in food. Plant polyphenols in the diet have a plethora of health benefits [6]. These compounds can prevent the negative reactivity of undesired reactive oxygen/nitrogen species produced by metabolic activities in the body. Because of their potential health benefits, polyphenols and other dietary phenolics are generating much attention in the scientific world.

Inflammation is a biological response when tissue homeostasis is disrupted by a natural, synthetic, or foreign agent [7]. Pathogens (parasites, fungi, and microorganisms), stress (shock or burns), and toxic compounds can all trigger an immune system response [8]. During infections, microorganisms and macrophages can stimulate the generation of pro-inflammatory mediators, such as interleukin IL-1, IL-6, and IL-8, reactive oxygen species (ROS), nitric oxide, and prostaglandins. Chronic infection, resulting in the excessive release of pro-inflammatory factors, has been associated with the development of degenerative conditions, including arthritis, atherosclerosis, asthma, Alzheimer’s disease (AD), and malignancies [9].

AD is the most prevalent neurodegenerative disease, accounting for 60–70% of dementia cases [10,11]. Approximately 44 million people are estimated to have been diagnosed with AD or associated dementia to date, and this population is expected to grow to over 135 million by 2050 [12,13]. The primary neuropathological hallmarks of AD are proteinaceous aggregates, such as intracellular neurofibrillary tangles containing hyperphosphorylated tau and extracellular senile plaques containing amyloid-beta deposits of varying lengths [14,15,16,17], resulting in neuronal cell death and degeneration associated with memory loss and severe cognitive impairment, disrupting activities of daily living [18,19,20,21]. In addition to protein aggregation, AD is associated with a neuropathological level of glial activation and neuronal cell death [22,23,24,25,26]. Environmental, lifestyle, and genetic risk factors can contribute to AD development [27,28,29].

Hypertension is a common and often progressive condition associated with a high risk of cardiovascular disease and related complications [30]. Hypertension is a critical health problem, and the incidence of hypertension is increasing globally, with some estimates suggesting that hypertension affects one-fourth of the total adult population worldwide [31]. The inhibition of the angiotensin-converting enzyme (ACE), which converts angiotensin I to angiotensin II, has been used to treat inflammatory disease (ID) and has demonstrated powerful antihypertensive effects [32]. 

Sugar uptake issues can lead to diabetes mellitus (DM), obesity, and oral diseases [33]. One of the most critical ongoing issues is the increased incidence of DM, caused by hyperglycemia, which describes an increase in the circulating blood glucose concentration. Two types of DM have been identified: type I, which is caused by insufficient insulin production, and type II, which is the result of insulin inefficiency or also insufficient insulin production [34]. Mammalian skin tone is prevalently determined by the level of melanin, which is a pigment responsible for protecting the skin from ultraviolet (UV) damage. The melanin content is related to glycemic control of diabetes and obesity. The lower the melanin content, the higher possibility of microangiopathy [35].

In recent years, epidemiological examinations have revealed a critical increase in melanoma, especially among the white populace [36,37,38]. Polyphenols can be helpful to delay detrimental effects in neuronal, behavioral, age-related diseases due to their high antioxidant activities. One study examined the antioxidant capacity of grape seed extract (GSE) in different regions of the central nervous system (CNS) in young and old rats [39]. Reducing the inflammatory response is often critical for many disease states, and non-steroid anti-inflammatory drugs (NSAIDs) are often used for this purpose. However, NSAIDs are often associated with adverse side effects, including gastrointestinal complications, water retention, kidney deficiency, bronchospasm, and hypersensitive responses [40,41].

Phenolic and polyphenolic products, either alone or in combination with vitamins, such as carotenoids, vitamin E, and vitamin C, act as antioxidants that protect the tissues in the human body from the damaging effects of oxidative stress. Polyphenols are the most common antioxidants found in fruit and vegetable-based diets. Gallic, ellagic, protocatechuic, and 4-hydroxybenzoic acids are the most common benzoic acids consumed by humans, whereas caffeic, ferulic, sinapic, and *p*-coumaric acids are the most common cinnamic acids. Plant-based diets are high in polyphenols, which provide nutritional advantages and contribute to preventing chronic diseases [42].

The current level of knowledge about the biochemical effects of dietary polyphenols and their involvement in human health and disease is discussed in this study. This study aims to describe the multifunctional roles of phenolic compounds in the treatment of human diseases. Phenolic compounds have been used for many therapeutic purposes due to their effects on inflammation and other characteristics of human diseases, which may guide future research. 

## 2. Health Benefits of Phenolic Compounds

**Figure 1:**
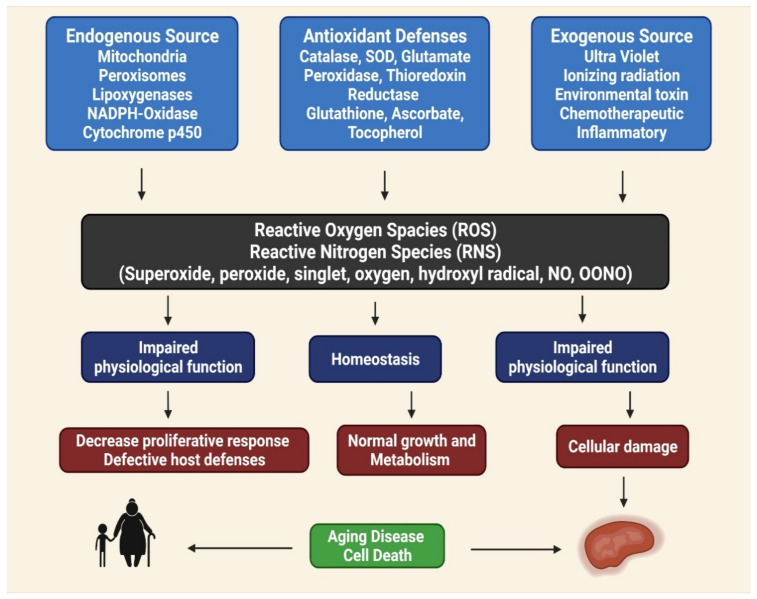
Role of reactive oxygen (ROS) and reactive nitrogen (RNS) species for aging disease and cell death, homeostasis and impaired physiological function [48].

Phenolic compounds can be found in various fruits and vegetables, especially grapes, berries, and tomatoes. Phenolic compounds can benefit one’s health by reducing the risks of developing metabolic disorders, such as type 2 DM [33]. The biological properties of phenolic compounds are diverse, although the specific mechanisms they exert their disease preventive effects remain unknown. Antioxidants contribute to the removal of these oxidative products. Under normal cellular circumstances, ROS and reactive nitrogen species (RNS) are incredibly reactive molecules; for example, ROS and RNS can disrupt mitochondrial respiration, damaging critical biological macromolecules, including proteins and DNA [43,44,45]. The anti-inflammatory, anti-aging, antiproliferative, and antioxidant properties of phenolic compounds have been described in several studies. In totaling the upstairs modifications, antioxidant enzymes are crucial for preventing oxidative damage [46]. Reactive oxygen (ROS) and reactive nitrogen (RNS) species are highly reactive oxidized molecules, including superoxide, peroxide, singlet oxygen, hydroxyl radical, nitric oxide (NO), and peroxynitrite (OONO^−^), that are constantly produced under normal cellular conditions, such as during homeostasis, impaired antioxidant functions can lead to cellular damage, resulting in aging, disease, and cell death (Figure 1) [47].

In intact cell systems, photo-activated reactive oxygen species (ROS) may activate the mitochondrial permeability transition pore (mPTP) within individual mitochondria. After the occurrences of ROS-triggering of the mPTP followed by additional stimulation of ROS production, the phenomenon of ROS-induced ROS release (RIRR) [49] was named. mPTP opening is a mitochondrial response to oxidative stress that causes an increase in ROS signal, which can have various consequences depending on ROS levels. In addition to the effects of ROS in those mitochondria, ROS released into the cytosol could stimulate a complicated cellular signaling response and RIRR in neighboring mitochondria (where the RIRR originated). In the latter case, ROS trafficking between mitochondria could serve as a positive feedback mechanism, increasing ROS production that spreads throughout the cell and causes visible mitochondrial and cellular damage. Although photo-induced ROS formation in the laboratory could be used to trigger more massive, avalanche-like ROS release, this phenomenon represents a more fundamental mechanism, such as light-independent spontaneous redox transitions associated with the induction of mPTP or another mitochondrial channel(s) that may occur under various physiological or pathological conditions. This review will cover a broad spectrum of physiological and pathological RIRR-related processes, such as mitochondrial ROS production and scavenging. Finally, an imbalance in the intake, neutralization, and outflow of ROS and associated triggers in particular cell signaling pathways can lead to oxidative and reductive stressors, which can cause a range of illnesses or even cell organismal death [50].

## 3. Bioavailability of Phenolic Compounds

The primary sources of phenolic compounds are fruits [51], vegetables, and beverages, such as coffee, tea, wine, and fresh fruit juices. Although Coffee is known for its stimulating properties attributed mainly to caffeine, it also contains other biologically active compounds, including phenolic compounds, with chlorogenic acids being the most abundant. The critical factor that affects these compounds in green coffee is the roasting time–temperature profile [52]. Polyphenols found in green tea include, but are not limited to, epigallocatechin gallate (EGCG), epigallocatechin, epicatechin gallate, and epicatechin; flavanols such as kaempferol, quercetin, and myricetin are also found in green tea [53]. In addition to exploring the potential protective effects, these compounds provide health benefits against chronic diseases, understanding the modifications during food processing techniques and, therefore, overall bioavailability is essential.

The bioavailability of bioactive compounds is the absorptive process of these molecules across the intestine into the circulatory system, after food ingestion. Several polyphenols can be ingested as either purified, isolated substances or in foods. Following the intake of polyphenols ranging from 6.4 to 1000 mg/day, detectable plasma levels ranged from 0.072 to 5 µM [54]. The polyphenol intake measured for an older Japanese population was reported to range from 183 to 4854 mg/day, with average information of 665–1492 mg/day. Coffee and green tea were the most common sources of these compounds [55].

**Figure 2:**
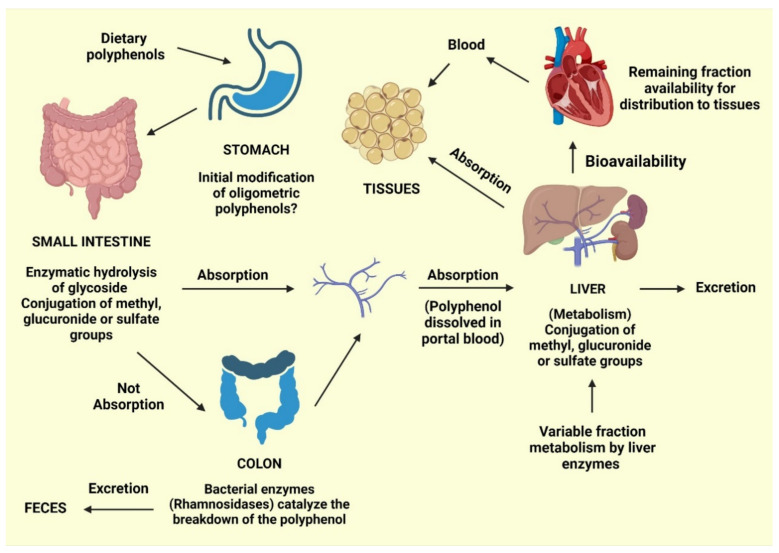
Predicted routes for the absorption of dietary phenolics [42].

Phenolic acids typically constitute approximately one-third of the total phenolics consumed, whereas flavonoids comprise the remaining two-thirds. Phenolic and polyphenolic products, either alone or in combination with vitamins, such as carotenoids, vitamin E, and vitamin C, can serve as antioxidants to protect various tissues in the human body from oxidative stress. Polyphenols are the most common antioxidants found in fruit and vegetable-based diets. Gallic, ellagic, protocatechuic, and 4-hydroxybenzoic acids are the most common benzoic acids consumed by humans, whereas caffeic, ferulic, sinapic, and *p*-coumaric acids are the most common cinnamic acids. Plant-based diets are commonly high in polyphenols, providing nutritional advantages and protecting against the emergence of chronic diseases. However, food processing techniques, including blanching and thermal treatments, can alter polyphenol levels or induce conversion into secondary compounds. Enzymatic and nonenzymatic reactions can activate the absorption and metabolism of phenolics, in addition to molecular changes that might occur during food production (Figure 2). Conjugation reactions may also increase or decrease the bioavailability of these molecules [42].

During the absorption process, gastric acid from the stomach can cause initial modifications to oligomeric polyphenols. Following ingestion, glycosidic polyphenols are cleaved in the small intestine, releasing the glycoside radical. Lactase phlorizin hydrolase and cytosolic glucosidase are enzymes with an affinity for glucose, xylose, and galactose [56].

However, polyphenols that are not cleaved by these enzymes are not absorbed by the small intestine and can be cleaved into small molecules known as phenolic acids produced by intestinal bacteria. Polyphenol structures can also be involved in conjugation reactions, resulting in methyl, glucuronide, or sulfate groups. The remaining polyphenols, especially those attached to rhamnose, can be processed by rhamnosidase released by the colonic microbiota.

Following these absorptive processes, phenolics will typically follow one of four paths: (1) Excretion in the feces; (2) absorption by the mucosa of the intestines or the colon, followed by entry into the portal vein for delivery to the liver; (3) further conjugation in the liver can result in the addition of with methyl, glucuronide, or sulfate groups, followed by release into the bloodstream for tissue absorption; and (4) excretion in the urine [42]. Bioavailability refers to the percentage of a nutrient that is digested, absorbed, and metabolized normally. The bioavailability of each polyphenol varies, but there is no link between the number of polyphenols consumed and their bioavailability in the human body. Although aglycones can be absorbed through the small intestine, most polyphenols found in food are present as esters, glycosides, or polymers, which cannot be absorbed in their natural state [57]. Before these compounds may be taken, they must be processed by intestinal enzymes or colonic microbes. Polyphenols undergo significant changes during absorption; they are conjugated in intestinal cells and later in the liver by methylation, sulfation, and glucuronidation [58]. As a result, the forms that reach the bloodstream and tissues differ from those found in food, making it difficult to identify and quantify all of the metabolites’ biological activity [59]. The chemical structure of polyphenols, rather than their content, determines the rate and amount of absorption and the type of metabolites circulating in the plasma. Because the most common polyphenols in our diet do not necessarily have the highest amounts of active metabolites in target tissues, polyphenol biological activities vary greatly. The increased plasma antioxidant capacity after consuming polyphenol-rich meals suggests that they are absorbed via the gastrointestinal barrier [60,61]. Polyphenols have distinct absorption locations in humans. Some polyphenols are absorbed well in the stomach, whereas others are absorbed more efficiently in the intestine or other regions of the digestive tract. All flavonoids in foods are glycosylated, except for flavanols. The fate of glycosides in the stomach is largely unknown. Most glycosides are unlikely to be hydrolyzed by stomach acid and hence reach intact in the intestine [62], where only aglycones and a few glucosides are absorbed. Some flavonoids, such as quercetin, can be absorbed at the stomach level, but not their glycosides, according to rat study [63]. Furthermore, anthocyanins are recently shown to be absorbed from the stomach in rats and mice [57,64]. According to one theory, glucosides are transported into enterocytes by the sodium-dependent glucose transporter SGLT1 and then destroyed by a cytosolic -glucosidase. Isoflavones, on the other hand, appear to be less affected by glucosylation than quercetin in terms of absorption [65]. Proanthocyanidins differ from most other plant polyphenols because of their polymeric shape and huge molecular weight. This feature should limit their absorption through the gut barrier, and oligomers larger than trimers in their native forms are unlikely to be absorbed in the small intestine [57,66]. When eaten in their free form, hydroxycinnamic acids are swiftly absorbed by the small intestine and conjugated into flavonoids [67]. Because the intestinal mucosa, liver, and plasma lack esterase capable of hydrolyzing chlorogenic acid to liberate caffeic acid, and hydrolysis can only be conducted by colonic microbiota. These chemicals are naturally esterified in plant products, and esterification hinders their absorption [68]. Even though the majority of polyphenols are absorbed in the gastrointestinal tract and intestine, some polyphenols are not. These polyphenols travel to the colon, where bacteria hydrolyze glycosides into aglycones, which are then converted into a variety of aromatic acids [69]. Aglycones are split at different points along the heterocycle depending on their chemical structure, resulting in different acids that are then metabolized to benzoic acid derivatives. Following absorption, polyphenols go through a variety of conjugation pathways. These activities generally consist of methylation, sulfation, and glucuronidation, which are metabolic detoxication processes that increase the hydrophilicity of xenobiotics and so assist biliary and urine excretion. Polyphenol methylation is also very specific; it normally occurs in the C_3_ position of the polyphenol, but it can also occur in the C_4′_ position: In fact, a large amount of 4′-methylepigallocatechin has been found in human plasma after tea drinking [70]. During the sulfonation process, sulfo-transferases catalyze the transfer of a sulfate moiety. Sulfation occurs largely in the liver, but the precise location of sulfation for polyphenols is unknown [71]. Glucuronidation occurs in the stomach and liver, with the largest rate of conjugation occurring in the C_3_ position [72]. Green tea catechins, whose aglycones can account for a significant portion of the overall amount in plasma [73], have highly efficient conjugation processes, and free aglycones are frequently absent or present in low amounts in plasma following dietary dosages. Because the nature and placements of the conjugating groups on the polyphenol structure can affect the biological properties of the conjugates, it is vital to identify the circulating metabolites, including their type and placements on the polyphenol structure. Polyphenol metabolites attach to proteins and circulate in the bloodstream; albumin is the most important protein involved in this process. In order for polyphenols to be bioavailable, albumin is required. The affinity of polyphenols for albumin varies according to their chemical structure [74]. The rate of metabolite elimination, as well as their distribution to cells and tissues, may be affected by albumin binding. It is possible that metabolite absorption is related to the concentration of unbound metabolites in the cell. Finally, whether polyphenols must be in their free state to have biological effect or if albumin-bound polyphenols might have biological activity is unknown [57]. Because this is the concentration at which polyphenols are biologically active for exerting their effects, the accumulation of polyphenols in tissues is the most important phase of polyphenol metabolism. Polyphenols have been shown in tests to permeate tissues, especially those that process them, such as the intestine and liver. In the urine and bile, polyphenols and their derivatives are eliminated. Highly conjugated metabolites are more likely to be excreted in bile, whereas small conjugates, such as monosulfates, are eliminated preferentially in urine, according to new research. The extent of metabolites removed in urine is roughly proportional to maximum plasma concentrations. The urine excretion % of citrus flavanones is high, but it decreases as you proceed from isoflavones to flavonols. As a result, polyphenols’ health advantages are reliant on their ingestion as well as their bioavailability [60].

## 4. Phenolic Compounds and Inflammation

Phenolic compounds are a heterogeneous group of secondary metabolites generated during plant metabolism. Due to their beneficial health effects, phenolic compounds have generated the interest of various experts, particularly their presence in foods. Phenolic compounds contain at least one aromatic ring to which one or more hydroxyl group is attached, and they may be aromatic or aliphatic. Flavonoids and non-flavonoids are two types of phenolic compounds [75].

### 4.1. Flavonoids

**Figure 3:**
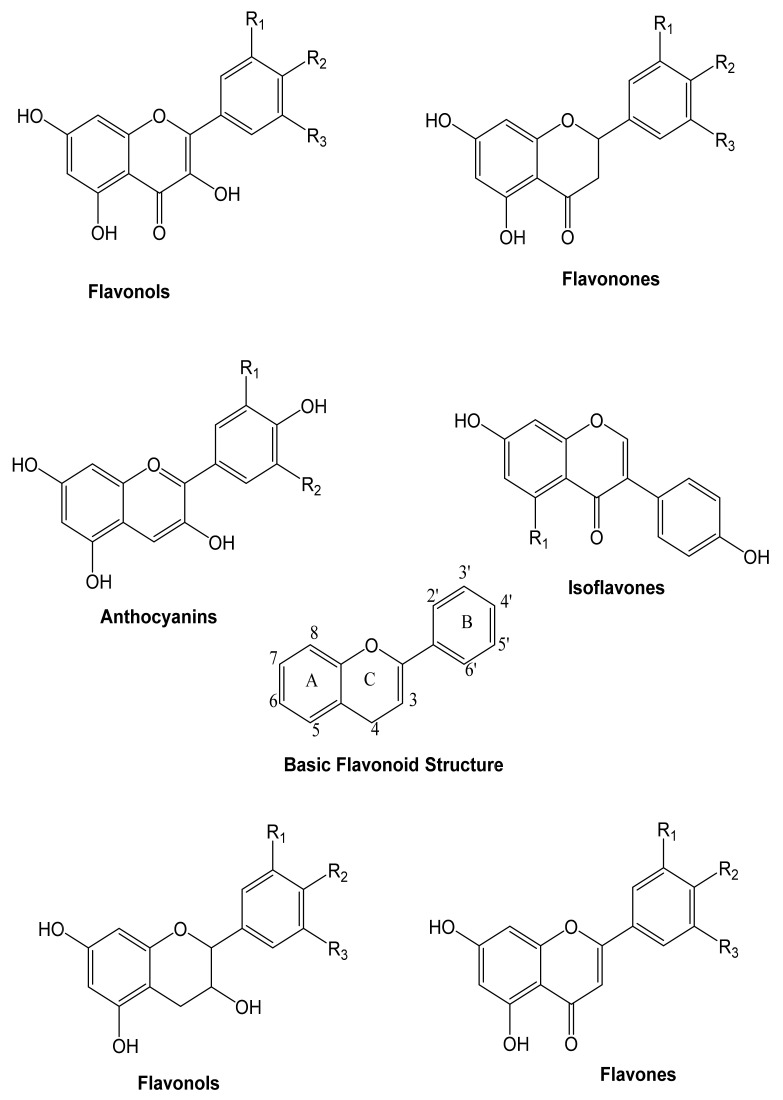
The general structure of the principal groups of flavonoids [76].

These are heterocyclic compounds consisting of two aromatic rings linked by an oxygen. Flavonoids can be divided into flavones, flavonols, anthocyanins, and isoflavones, depending on the hydrogenation status and the identities of heterocyclic substitution. Flavonoids are typically represented by glycosides (Figure 3).

### 4.2. Non-Flavonoids

**Figure 4:**
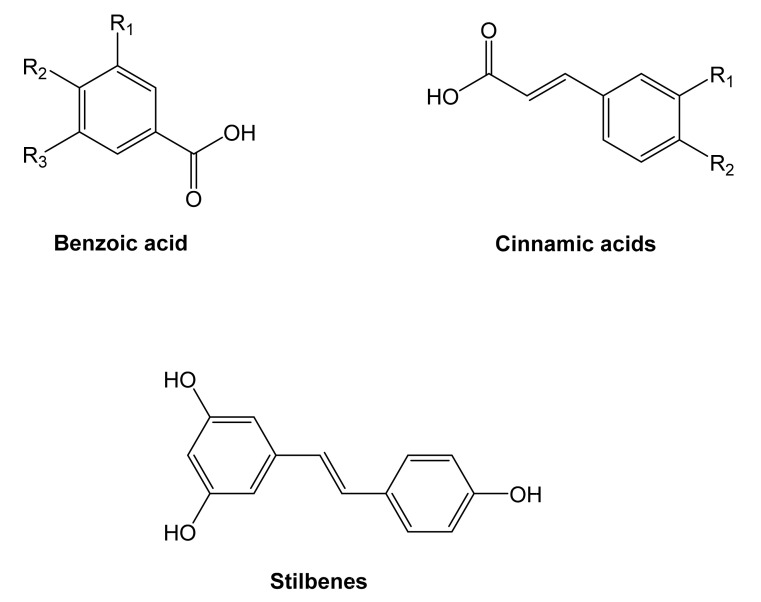
Structure of the principal non-flavonoids compounds [76].

Two of the most representative non-flavonoid compounds are benzoic and cinnamic acids, also identified as phenolic acids (Figure 4). Stilbenes, tannins, and lignins are derived from phenolic acids.

The absorption of phenolic fats (catechol, resorcinol, and hydroquinone) [77] from food products has been linked to the reduced occurrence of chronic illnesses, such as DM, cardiovascular disease, AD, Parkinson’s disease (PD), and infection, according to epidemiological data [45,47]. Phenolic compounds are considered to be responsible for these beneficial effects. Prolonged and severe illnesses have been associated with the development of chronic conditions, as described. As a result, invasive events that alter the inflammatory cascade associate with disease development may represent potential targets for disease prevention [78,79,80]. Some phenolic compounds have been shown to have anti-inflammatory properties. Although the specific mechanisms that underlie these anti-inflammatory activities are not yet understood, there is an element between a high absorption of these intensifies and a devaluation in intemperate response [81]. The relationship between phenolic complexes and anti-inflammatory activities has been examined, and the following criteria have been established based on the observed reactions with various inflammatory targets [53,54].

(i) For flavonoid molecules to be active, they must have a planar ring structure. (ii) The C ring must be unsaturated, due to the presence of a ketone carbonyl at C_4_ or a double bond between C_2_ and C_3_, for example. (iii) OH groups must be conjugated to the B ring and at C_5_ and C_7_ of the A ring. (iv) Flavones and flavones featuring an OH group at the 4′ position of the B ring had more significant activity than those without an OH group. (v) The activity improved following the methylation of the OH groups at positions 3, 5, or 4′. The methylation of the 3-OH group reduced (vi) Cytotoxicity. (vii) Flavones had more incredible activities than isoflavone, and flavonoids, although flavones are a type of flavonoid. (viii) Because aglycones are not glycosylated, they have more potent effects than glycosides.

The role of glycosides remains under debate because they have occasionally been shown to reduce anti-inflammatory activity [82] but facilitate absorption [83]. The relationship between the phenolic system and pro-inflammatory intermediaries has been demonstrated in further specialized applications. Flavonoids can inhibit NO due to three structural characteristics: (A) the C_2_=C_3_ double bond; (B) an unwieldy substituent group can either decrease inhibition by several-fold (aglycones have a stronger inhibitory effect than glycosides); and (C) 7 and 4′ OH groups can also affect inhibition, although to a lesser extent than the other characteristics [84].

## 5. Anti-Inflammatory Agents: Mode of Action of Phenolic Compounds

**Figure 5:**
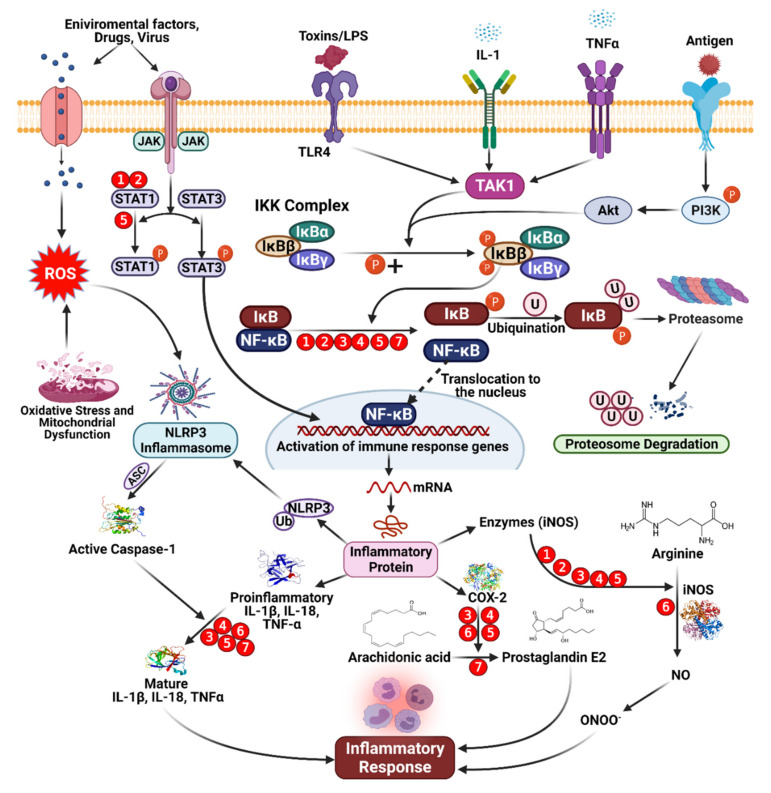
Mechanism of anti-inflammatory activity mediated by dietary phenolic compounds. Red circles indicate inhibition, and the numbers refer to the following phenolic compounds. (1) Genistein [84,93], (2) daidzein [84], (3) isorhamnetin [89], (4) pelargonidin [91], (5) kaempferol [92], (6) apigenin [90], and (7) epicatechin [93].

Phenolic compounds tend to act in a complementary manner with NSAIDs, but some phenolic compounds can also inhibit pro-inflammatory mediators’ activity or gene expression, such as cyclooxygenase (COX). Phenolic compounds can also up- or downregulate transcriptional elements involved in antioxidant pathways, such as nuclear factor-κB (NF-κB) or nuclear factor-erythroid factor 2-related factor 2 (Nrf-2) [59,85]. The structures of phenolic compounds can significantly impact their anti-inflammatory mechanisms. By resonance, unsaturation in the C ring, for example, appears to affect the strength of binding interactions. Furthermore, the formation of a double bond between C_2_ and C_3_ causes coplanarity between the A and C rings, which increases the interaction between flavonoids and synthetic active sites [86]. Enzymatic activity inhibited by catechols improves depending on the structure of the B ring, which relies on the development of electrophilic domains and requires nucleophilic additions. Phenolic ligands can enhance the formation of covalent bonds between flavonoids and macromolecules [81]. Phenolic compounds are thought to suppress the binding of pro-inflammatory mediators, regulate eicosanoid synthesis, inhibit stimulated resistant units, or inhibit the activity of NO synthase and COX-2 through inhibitory effects on NF-κB [52,60,62]. Inflammatory mediators, such as IL-6, are affected by dietary flavonoids, such as flavones found in cocoa and tea, which have a dose-response effect on IL-6 levels in the blood [87]. Some subjects demonstrated a positive impact on the reduction in inflammatory markers. However, the mechanism linking the absorption of phenolic compounds from cocoa and the resulting levels of inflammatory markers (IL-1, 1L-6, tumor necrosis factor [TNF]-α), but a reduction in low-density lipoprotein (LDL) was reported, which could result in reduced vascular inflammation, oxidative stress, and NO levels and the prevention of platelet aggregation, reducing the risk of heart disorders [49,64]. Extensive reviews describing the anti-infection properties of phenolic compounds have again been directed toward us and red flame consumption. Grape phenolic extracts have been used for both in vitro and in vivo experiments. Procyanidins have been shown to inhibit inflammatory mediators, resulting in reduced concentrations of NO, prostaglandin E_2_, and ROS. The antioxidant properties of phenolic compounds were primarily responsible for these effects [88,89,90,91,92]. The inhibitory activities of pro-inflammatory intermediaries or changes in gene expression are involved in the impact exerted by phenolic compounds (Figure 5). An increase in the level of phenolic compounds or multiple phenolics may have anti-inflammatory effects through various pathways, whereas synthetic molecules can typically only affect one component (Table 1).

**Figure 6:**
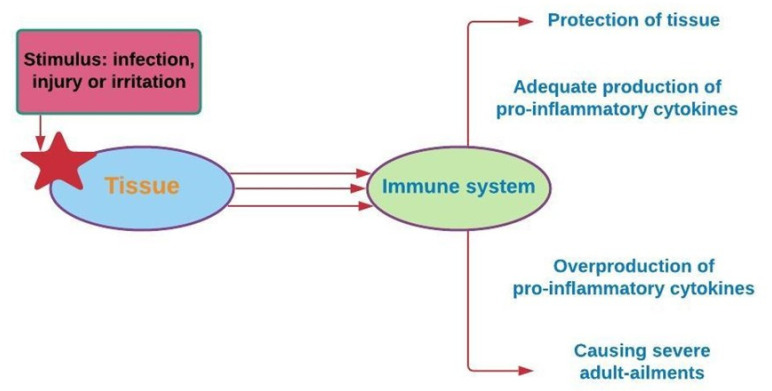
The occurrence of chronic diseases is associated with the overproduction of pro-inflammatory cytokines [102].

Inflammation is a biochemical reaction to tissue injury that is essential for survival. The immune system responds to stimuli such as infection, injury, or irritation through the release of pro-inflammatory cytokine [99]. The overproduction of pro-inflammatory cytokines, including IL-1b, IL-6, and TNF-α, leads to severe illnesses among adults, including asthma, atherosclerosis, allergies, and cancer (Figure 6) [100].

Preventing inflammation-associated diseases requires the inhibition of pro-inflammatory cytokine overproduction. Since ancient times, phytochemicals derived from plant-based formulations have been widely used to treat inflammation and associated disorders. Among identified phytochemicals, phenolics are essential for suppressing inflammation, and recent research has revealed their potent anti-inflammatory properties. Pragasam et al. [101] investigated the anti-inflammatory efficacy of *p*-coumaric acid by measuring the expression of the inflammatory mediator TNF-α in the synovial tissue of adjuvant-induced arthritic rats. They discovered that *p*-coumaric acid has a potent anti-inflammatory function through the reduction of TNF-α expression.

## 6. Phenolic Compounds as Inhibitors of Enzymes Associated with Human Disease and Other Roles in Human Diseases

Prescription medications are often designed to act as inhibitors of compounds involved in disease mechanisms. Concerns regarding harmful adverse effects caused by engineered catalyst inhibitors have prompted the search for new effective and protective inhibitors derived from natural sources. Plant phenolic compounds are among the most commonly explored groups due to their enormous scope of biological effects. This section provides an overview of known phenolic compounds with protein inhibitory activity. To examine the chemical inhibitory capacities of phenolic compounds against various proteins known to be involved in severe human conditions, wide-reaching research has been conducted [103]. Catalysts are perhaps the best focal point for identifying drug-based inhibitors for treating human diseases because they are significantly involved in several physiological cycles [104]. The utilization of compound inhibitors found in specific fundamental human food sources has been examined, which have reported effects on hypertension, metabolic issues, incendiary infections, and neurodegenerative diseases. Several food sources containing identified inhibitors have been reported in treating a variety of symptoms, including those associated with hepatotoxicity, gastrointestinal problems, and diarrhea [76,77].

### 6.1. Hypertension: Inhibition of Angiotensin-Converting Enzyme (ACE)

**Figure 7:**
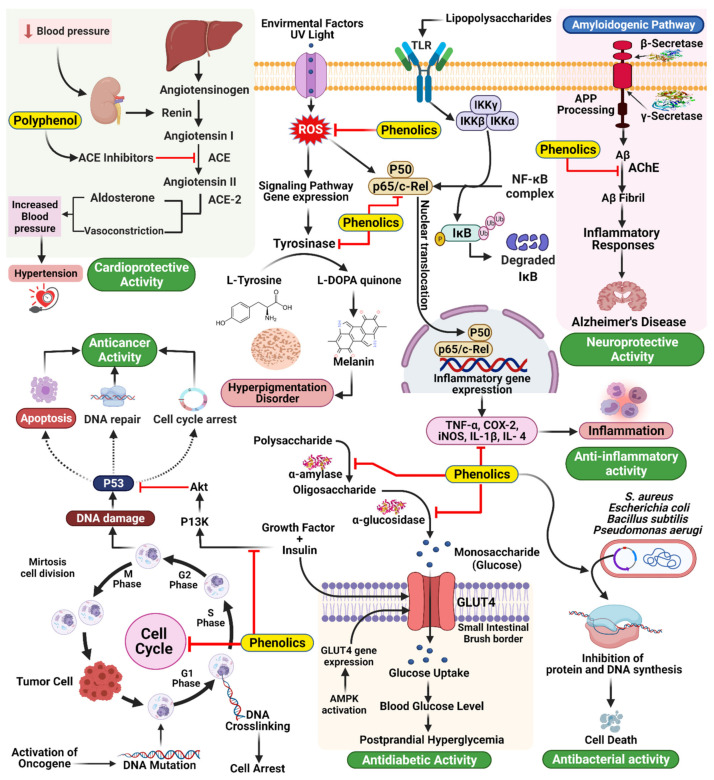
Mechanism of action underlying the therapeutic activities of phenolics in different diseases.

Hypertension is a common and often progressive condition associated with a high risk of cardiovascular disease and related complications [30]. Hypertension is a critical and increasingly common health problem worldwide, and estimates suggest that up to one-quarter of the global adult population suffers from hypertension [31]. ACE inhibition is a common goal for treating ID and has been shown to have antihypertensive effects [32]. ACE catalyzes the conversion of angiotensin I into angiotensin II, a vasoconstrictive peptide, and degrades bradykinin, a potent vasodilator [105]. Common ACE inhibitors, including captopril, benazepril, and enalapril, among others [106], have been associated with adverse effects, including an overall reduction in proteolytic activity. Consequently, attempts have been made to identify novel ACE inhibitors from natural sources, mainly plant sources. Many studies have shown that food sources rich in polyphenols are powerful for preventing and treating hypertension, specifically through ACE inhibition (Figure 7) [107]. In a new report, 74 plant families with significant ACE inhibitory action were distinguished by Patten et al. [108]. Field and Newton [109] have similarly demonstrated that cocoa polyphenols (catechins, flavonol glycosides, anthocyanins, procyanidins) are bioavailable particles with antihypertensive activity through ACE inhibition [110].

### 6.2. Type 2 Diabetes Mellitus: Inhibition of Carbohydrate Hydrolyzing Enzyme

Using an in vitro model, Bhandari et al. studied the antidiabetic efficacy of *Bergenia ciliata*. The active chemicals (−)-3-*O*-galloylepicatechin and (−)-3-*O*-galloylcatechin, found in the ethyl acetate soluble *B. ciliata* extract, were found to be responsible for the substantial inhibition of porcine pancreatic-amylase and rat intestinal maltase activity in a dose-dependent manner [111]. The half-maximal inhibitory concentration (IC_50_) value, often used to quantify inhibitor potency, is defined as the concentration of an inhibitor necessary to inhibit enzyme activity by 50%. The IC_50_ values for (−)-3-*O*-galloylepicatechin were 334 and 739 M for rat intestinal maltase and porcine pancreatic-amylase, respectively, and those for (−)-3-*O*-galloylcatechin were 150 and 401 M, respectively [111]. The inhibitory activities of these compounds against α-glucosidase and α-amylase in vivo and in vitro demonstrated that they have an excellent potential for development as a treatment for type 2 DM [112].

Delaying glucose absorption through inhibiting -glucosidase activity is one potential treatment option for type 2 DM prevention. *Terminalia chebula* fruits contain phenolics that present significant enzymatic inhibitory activity against mammalian-glucosidases, which could contribute to the management of blood glucose levels in patients with type 2 DM without causing severe adverse effects Quercetin, kaempferol, luteolin, quercetagetin, and scutellarein are naturally occurring flavonoids that function as inhibitors of human α-amylase, making them intriguing candidates for limiting starch digestion (Figure 7) [34].

Natural health substances including non-flavonoid polyphenols (e.g., resveratrol, curcumin, tannins, and lignans), flavonoids (e.g., anthocyanins, epigallocatechin gallate, quercetin, naringin, rutin, and kaempferol), plant fruits, vegetables, and other products (e.g., garlic, green tea, blackcurrant, rowanberry, bilberry, strawberry, cornelian cherry, olive oil, sesame oil, and carrot) may be a safer alternative to primary pharmacological therapy. Flavonoids are essential components of the human diet, and they may contribute to DM management by delaying the deterioration of pancreatic beta-cell function caused by oxidative stress. They are recommended as food supplements to prevent and ameliorate T2DM-related complications [113,114]. 

### 6.3. Skin Hyperpigmentation: Inhibition of Tyrosine

Mammalian skin tone is prevalently dictated by the levels and locations of the pigmentation melanin. Melanin protects the skin from the harmful effects of UV light; however, melanin overproduction can result in the development of various dermatological problems. For example, melasma and age spots are caused by the abnormal accumulation of epidermal pigmentation [115]. Three reactions in the biosynthetic cycle of melanin in melanocytes are catalyzed by tyrosinase. Tyrosine is first hydroxylated to L-DOPA and then oxidized to dopa quinone. Following a series of oxidoreduction reactions, the moderate dihydroxy indole (DHI) and dihydroxy indole carboxylic corrosive are generated and polymerize to frame melanin [116]. Tyrosinase inhibition is one of the key strategies used to treat hyperpigmentation; however, concerns regarding the toxicity and adverse effects of synthetic inhibitors have prompted the search for new protective and effective tyrosinase inhibitors derived from natural products. Tyrosinase is often used as a soil-conditioning agent, and inhibitors of this compound are often added to plant-based nourishments. For the development of skin-brightening agents, similar to pest control substances, the identification of tyrosinase inhibitors is fundamental. Various scientists have attempted to identify inhibitors from natural sources, such as plants. Phenolics and polyphenols represent the largest group of phytochemical substances with dynamic tyrosinase inhibitory activity (Figure 7) [117]. The largest group of newly identified natural tyrosinase inhibitors are flavonoids, and their structures contain elements similar to those of tyrosinase substrates and known tyrosinase inhibitors [117,118]. Steppogenin, for example, is a flavanone derivative isolated from *Cudrania tricuspidata*, which showed significantly more decisive inhibitory action against tyrosinase than kojic acid, a known tyrosinase inhibitor [119]. Some stilbenes have also been recognized as having tyrosinase inhibitory and degradation properties, including resveratrol, oxyresveratrol, and chlorophorin [118,120,121].

### 6.4. Inflammation: Inhibition of Pro-Inflammatory Enzymes

Numerous ailments are associated with chronic aggravation, including DM, obesity, malignancy, osteoarthritis, atherosclerosis, and Crohn’s disease. The aggravation systems remember a grouping of occasions for which arachidonic acid digestion plays a significant role in catalyzing the conversion of arachidonic acid to prostanoids by COX-1 and COX-2. 5-Lipo oxygenase (LOX) is involved in a second pharmacologically practical metabolic pathway for arachidonic acid, resulting in the biosynthesis of leukotrienes, a class of inflammatory mediators. Pro-inflammatory compounds include COXs, which affect platelet aggregation, vasoconstriction, vasodilatation, and LOXs, affecting atherosclerosis development [122,123]. Much attention has been paid to the two COX proteins and 5-LOX because they have been identified as putative malignancy prevention targets. Nonsteroidal and steroidal anti-inflammatory drugs exert their activity by inhibiting these pro-inflammatory mediators through various mechanisms [124]. Although current anti-inflammatory agents can inhibit intense inflammatory responses, unfavorable effects are associated with the continued use of these medications to combat chronic inflammatory states. These adverse effects legitimize the search for new and safe inflammatory mitigating agents derived from plant compounds. Recently, interest in flavonoids’ inhibitory and immunomodulatory capability has increased, including their capacity to inhibit pro-inflammatory secretion [125,126,127]. Polyphenolic compounds inhibit phosphatidylinositide 3-kinases/protein kinase B (PI3K/AkT), inhibitor of kappa kinase/c-Jun amino-terminal kinases (IKK/JNK), mammalian target of rapamycin complex 1 (mTORC1) which is a protein complex that controls protein synthesis, and JAK/STAT. They can suppress toll-like receptor (TLR) and pro-inflammatory genes’ expression. Their antioxidant activity and ability to inhibit enzymes involved in the production of eicosanoids contribute as well to their anti-inflammation properties. A series of in vitro studies found that polyphenols like oleanolic acid, curcumin, kaempferol-3-*O*-sophoroside, EGCG and lycopene inhibit high mobility group box1 protein, an important chromatin protein that interacts with nucleosomes, transcription factors, and histones regulating transcription and playing a key role in inflammation. All of these examples support the anti-inflammatory effects of polyphenols [128].

### 6.5. Alzheimer’s Disease (AD): Inhibition of Cholinesterase

AD is a progressive, age-related neurodegenerative disease that represents the most predominant form of dementia. The rapidly aging human population has increased the incidence of AD worldwide, and global AD rates are projected to improve immensely, particularly in developing regions. Although the exact pathogenesis of AD has yet to be clarified, it is currently believed to be a multifactorial disease. Postmortem research performed during the mid-1970s showed that choline uptake and acetylcholine levels were diminished in the cerebrums of AD patients, which was associated with severe presynaptic cholinergic deficits [129]. This finding prompted the cholinergic-deficiency hypothesis, which suggests that a disruption in the cholinergic capacity is an underlying factor in AD development, associated with deficits in learning, memory, behavior, and excitatory reactions in various cerebral regions, including the neocortex and the hippocampus. Acetylcholine is rapidly hydrolyzed by acetylcholinesterase (AChE) [96,97], and acetylcholine levels at the synapse are responsible for the conduction of electrical impulses that transmit from one neuron to the next, which becomes diminished under conditions of acetylcholine deficiency. Butyryl cholinesterase (BChE) is a catalyst firmly identified with AChE and serves as a co-regulator of acetylcholine hydrolysis and cholinergic neurotransmission [130]. During AD progression, some studies have shown expanded activity of BChE in the most affected brain regions. The inhibition of both AChE and BChE increases acetylcholine availability and diminishes amyloid-beta accumulation, significant AD features. BChE expression is primarily restricted to the fringe tissues, with only small quantities of BChE found in the primary cerebral cortex. The potential advantages of the specific inhibition of AChE without inhibiting BChE could result in reduced responses due to the remaining cholinesterase activity in the fringe regions [131].

However, the mechanisms through which polyphenols act on important cellular events have not been completely elucidated to date. Phenolic compounds interact with the amino acid residues that define the active site of AChE via the formation of hydrogen bonds and hydrophobic and π–π interactions [132]. Multiple hydroxyl groups in the phenolic compound are thought to enhance the inhibition of AChE due to the enhanced binding capacity [133]. These inhibitory activities explain the functional potential associated with most phenolic compounds, but not all act through the exact mechanism [134]. The role of different polyphenols and their modes of action are shown in Table 2. 

### 6.6. Activity of Phenolic Compounds in Skin Diseases

The skin is an essential organ of the human body, representing the largest surface area and directly contacting the environment. Various skin diseases can undermine our lives, such as malignancy (Table 3) [142].

### 6.7. Skin Cancer

The most dangerous skin diseases are skin tumors, including squamous cell carcinoma, basal cell carcinoma, and threatening melanoma. Although threatening melanoma is less common than the other two types, it is often the most serious. Melanoma can be treated adequately through a simple medical procedure during the early phases; however, melanoma is often associated with a high death rate due to high levels of metastasis and poor response to chemotherapy. In recent years, epidemiological examinations have shown a critical expansion in the occurrence of melanoma, especially among the white populace [36,37,38]. High death rates among melanoma patients have prompted numerous scientists to search for effective feature-based treatments. Spices and medications derived from plants have long been used to treat tumors and are increasingly used in modern society [157,158]. Among the currently available anticancer drugs, 60% depend on natural compounds and their metabolites. It has increased interest and trust in biological agents, which represent significant hotspots for developing viable therapeutic agents for various diseases [159].

Phenolic compounds that can affect the cell cycle represent promising natural compounds that inhibit malignant growth, such as skin tumors. Curcumin, which is a well-known apoptotic compound, is one possible compound. Studies have shown that p53 is not activated by curcumin, which is significant for treating p53-transformed melanomas that are impervious to regular chemotherapy. Curcumin activates caspase-3 and caspase-8 but not caspase-9, and, through a layer intervened system, apoptosis happens [160,161,162]. The chemopreventive effects of polyphenols as anthocyanins, ellagitanins, EGCG, oleuropeindihydroxy phenyl, punicalagin, quercetin, resveratrol and theaflavin, were mainly examined in treatment of melanoma as the highly metastatic form of the cutaneous cancer. These polyphenols are mediated by several signaling pathways against skin carcinogenesis and metastasis, implying the importance of polyphenols to open up new horizons in development of anti-skin cancer therapeutic strategies [163].

### 6.8. Psoriasis

Psoriasis is a hereditary disease characterized by cutaneous irritation, expanded epidermal growth, hyperkeratosis, angiogenesis, and strange keratinization, with a T cell infiltration into the inflamed skin tissue [162,164]. Psoriasis is often characterized by dry or red patches of skin, coated with gleaming scales and red lines, and can affect various regions of the body, and all areas of the skin can be affected. Psoriasis often presents in the fingernails and toenails, on the skin of the trunk, elbows, knees, and scalp. Skin injuries, breaks in the skin, aggravation, scratching, joint pain, increased irritation of the eyes, and small flaky skin spots on the skin, particularly among infants, are additional symptoms. Irritation and angiogenesis correspond with the pathophysiology of psoriasis, promoting uncontrolled keratinocyte outgrowth. To date, psoriasis, alongside natural elements, is solidly distinguished as a solid, unique hereditary background [165,166]. Phototherapies and medicines with antiproliferative effects that inhibit keratinocyte growth are the primary treatment options for psoriasis [125]. However, existing treatments can aggravate symptoms and induce phototoxicity, excessive sensitivity, organ damage, malignant growth, and systemic immunosuppression, and the identification of natural treatment options represents a vital alternative [166]. Immunosuppressive and growth mitigation effects against psoriasis have been demonstrated by various natural compounds, including polyphenolic compounds [167]. Psoriasis is a stable, recurring skin disease that affects up to 2% of the global population, characterized by well-defined macroscopic skin changes [168]. The development of psoriatic lesions involves two diverse cell types, mononuclear leukocytes, and epidermal keratinocytes. Keratinocytes produce factors that enable T cells and antigen-presenting cells to communicate directly [169]. Dietary activities have been linked to reductions in the clinical course and incidence of psoriasis in evidence-based clinical studies [170]. Dietary alterations can also diminish the incidence of side effects associated with the use of immunosuppressive drugs; from a pharmacoeconomic perspective, a well-balanced diet can lessen the costs of chronic disease care while also lowering the risks of complications [171]. It has been demonstrated that phytomedicine, which is used for psoriasis patients, provides some advantages, including natural sources, a lower risk of adverse effects, and the avoidance of dissatisfaction with conventional therapy. The herbal products’ structural diversity and multiple mechanisms of action have enabled the synergistic activity to mitigate psoriasis through the inhibition of keratinocyte-proliferation [172]. There are several modern drugs available for the treatment of psoriasis from polyphenol-rich dietary active compounds (Table 4) [173].

### 6.9. Acne Vulgaris

The anaerobic bacterium *Propionibacterium acnes* plays a significant role in the pathogenesis of skin inflammation. Antimicrobial treatments applied to the treatment of skin rashes, rosacea, and other non-resistant infections may also prevent *Propionibacterium acnes* colonization. A minimal skin inflammatory response following the application of antimicrobial agents, such as erythromycin and antibiotics, has been reported, leading to a lack of satisfaction with treatment. Benzoyl peroxide (BPO) is an exceptionally viable antibacterial agent against *Propionibacterium acnes,* which has no known targeted treatment option to date. In a combined treatment for skin rashes, including antimicrobials, retinoids represent a fundamental component and may represent an alternative treatment option [179]. Flavonoids identified in *Eucalyptus maculata* extract [180] or *Terminalia arjuna* [181] include alpha-mangostin, the principal compound in mangosteen natural skin products, which demonstrated significant antimicrobial activity against *Propionibacterium acnes* strains [182]. Honokiol and magnolol (isolated from *Magnolia sp.*), in addition to gallic, caffeic, chlorogenic, ferulic, myricetin, and cinnamic acids, quercetin, apigenin, luteolin, and thymol, derived from wild watermelon leaves, are other phenolic compounds that have demonstrated antibacterial effects against *Propionibacterium acnes* [183,184].

### 6.10. Skin Allergies and Atopic Dermatitis

This makes the invulnerable framework incapable of adapting to allergens around us, and an ever-increasing number of people experience the adverse effects of skin hypersensitivities and atopic dermatitis. A polluted environment can disrupt nourishment due to an increase in synthetics and stress. The incidence of skin hypersensitivity has increased globally over the past 20 years. Changes in dietary patterns are thought to represent an ecological factor contributing to the unfavorable changes in the presentation of detrimental symptoms and increased sensitivity to various factors. Changes in diet have been demonstrated to prevent hypersensitive reactions and improve the manifestation of symptoms. Devereux et al. have performed a longitudinal study showing that the dietary intake of cell reinforcements and lipids during pregnancy and youth may be associated with a lower risk of hypersensitive skin diseases onset [185]. In another investigation, Shaheen et al. showed that asthma incidence and severity was negatively associated with apple and red wine consumption in a population-based case-control study in London, likely due to the protective impacts of flavonoids [186]. The clinical implications of specific flavonoid-rich vegan diets that feature fewer calories in adult patients with atopic dermatitis were examined in a different examination, which showed a reduction in disease severity and improved serological parameters [187].

### 6.11. Antibacterial Effects and Antiviral Activities

Some plant species produce a wide range of phenolic compounds. *Camellia assumica* and *Camellia sinensis* are familiar catechin sources. Green tea primarily consists of water and phenolic substances (flavandiol, flavanols, phenolic acid, and flavonoids), and catechins represent greater than 75% of the polyphenols found in tea leaves [188]. C_60_(OH)_44_ is active catechin, often used as a regulator in studies of fullerenes, and its hydroxylated compounds feature antimicrobial activity [189]. Alvarez-Suarez et al. investigated the presence of carotenoids, flavonoids, amino and ascorbic acids, proteins, and total phenolic compounds and explored their antimicrobial properties in a variety of Cuban honey. Honey displays antibacterial effects, which moderately effectively against *Bacillus subtilis* and *Escherichia coli* (Figure 7) **[190]**. Pinosylvin, piceatannol, and pinosylvin monomethyl ether, all stilbenes, have been proposed to feature antibacterial activities. Pinosylvin susceptibility was exceptionally high for *Listeria monocytogenes* [191]. When assessing the antibacterial activities of phenolic compounds isolated from tobacco leaf, the ability to inhibit proliferation in *Staphylococcus aureus*, *Escherichia coli* and *Bacillus subtilis* was measured [192]. The in vitro activities of seven antimicrobial agents, including ceftazidime, ciprofloxacin, tetracycline, sulfamethoxazole, trimethoprim, piperacillin, and polymyxin B, and six polyphenols (gallic acid, ellagic acid, protocatechuic acid, rutin, myricetin, and berberine) against five *Pseudomonas aerugi* varieties were tested, both individually and in combination [193]. Although their bactericide and fungicide properties have been extensively studied, few studies have examined how biopolymers may behave as carriers of antiviral compounds or how they interact with the constituents of edible films or coatings. Furthermore, edible antiviral coatings can be engineered to inactivate viruses, usually more resistant to treatments than bacteria [194]. For example, GSE and green tea extract are two plant substances with known antiviral activities that can be useful in the food industry [195,196,197,198].

### 6.12. Anti-Aging Effects

With increasing age, aging is defined as the accumulation of various harmful changes in cells and tissues, increasing the risk of disease and mortality. One of the most widely recognized hypotheses for understanding the mechanism of aging is the free radical/oxidative stress theory [199]. Even under normal circumstances, some oxidative damage occurs; however, as the efficiency of antioxidative and repair mechanisms declines with age, the rate of this damage increases [200,201]. The antioxidant capacity of plasma is connected to antioxidant food intake; it has been discovered that eating an antioxidant-rich diet can help to reduce the adverse effects of aging and behavior. Several studies have suggested that a combination of antioxidant/anti-inflammatory polyphenolic chemicals in fruits and vegetables could be effective anti-aging agents [202]. Brightly colored fruits, such as berry fruits, concord grapes, and grape seeds, are exceptionally high in anthocyanins, a subset of flavonoids. Fruit pigments called anthocyanins have been proven to have powerful antioxidant and anti-inflammatory properties and suppress lipid peroxidation and the inflammatory mediator’s cyclo-oxygenase (COX)-1 and -2. Fruit and vegetable extracts with high quantities of flavonoids, such as spinach, strawberries, and blueberries, have high total antioxidant activity. Dietary supplementation with spinach, strawberry, or blueberry extracts in a control diet was likewise helpful in restoring age-related deficiencies in the brain and behavioral function in elderly rats, according to the findings [46]. According to a new study, tea catechins have potent anti-aging properties, and drinking green tea rich in these catechins may help delay the onset of aging [203]. Polyphenols can also help reduce the adverse effects of aging on the neurological system and brain. The ability of dietary polyphenols to cross the blood–brain barrier (BBB), which tightly limits the entry of metabolites, nutrients, and medications into the brain, is critical for their relevance in protecting the aging brain. Resveratrol has been demonstrated to extend life expectancy continuously; its activity is linked to a condition known as calorie restriction or partial food deprivation. Resveratrol, a grape polyphenol, is a relatively new antiaging agent. The sirtuin class of nicotinamide adenine dinucleotide (NAD)-dependent deacetylases has been demonstrated to be an early target of resveratrol. In mammals, seven sirtuins have been discovered, with SIRT-1 thought to mediate the health and longevity benefits of calorie restriction and resveratrol [204]. Resveratrol improved insulin sensitivity, reduced IGF-1 expression, and raised the activity of AMP-activated protein kinase (AMPK) and peroxisome proliferator-activated receptor-c coactivator 1a (PGC-1a). When the mechanism was investigated, it activated forkhead box O (FOXO), which regulates the expression of genes involved in lifespan and stress resistance, as well as insulin-like growth factor binding protein 1 (IGFBP1) (IGFBP-1) [205]. Experiments have shown that resveratrol can lengthen the lifetime of yeast. Fruit fly *Saccharomyces cerevisiae Drosophila melanogaster*, *C. elegans* (nematode worm), and *Nothobranchius furzeri* (seasonal fish). Recently, quercetin has been linked to an anti-aging effect [206].

### 6.13. Anticancer Effects

Epidemiological evidence has demonstrated the remarkable health-promoting effects of phenolics against chronic ailments, including anticarcinogenic, anti-inflammatory, and antioxidant activities (Table 5). Flavonoids are the most common phenolic compounds, comprised of chalcones that contain three aromatic rings and fifteen carbons [207,208,209]. Other phenolic acids, such as ferulic, feruloyl-l-arabinose, and coumaric, have been studied in numerous cell lines for their anticarcinogenic potential [210]. Ferulic acid has an anticancer impact on MIA PaCa-2 cells through effects on the cell cycle, invasion, and apoptotic behavior (human pancreatic cells) [211]. Researchers examined the synergistic anticancer potential of ferulic acid and -tocotrienol against the proliferation of various cancer cells and discovered that, when used together, this combination inhibits the proliferation of DU-145 (prostate cancer), MCF-7 (breast cancer), and PANC-1 (pancreatic cancer) cells better than when used separately [212]. According to Choi and Park, ferulic acid suppresses homologous recombination during DNA repair and RAD51 (eukaryotic gene) production in breast cancer cells. Furthermore, when combined with veliparib therapy, ferulic acid showed significant chemotherapeutic efficacy [213]. By slowing the cell cycle progression of Caco-2 colon cancer cells, *p*-coumaric acid was able to protect against the development of colon cancer. In lung cancer cells, feruloyl-l-arabinose inhibited cell penetration, motility, and ROS generation. Furthermore, flavonoids have demonstrated excellent anticarcinogenic capacities, such as troxerutin, apigenin, kaempferol, and myricetin [214].

### 6.14. Role of Phenolic Compounds in Immune System–Promoting and Anti-Inflammatory Effects

#### 6.14.1. Impact of Phenolic Compounds on Rheumatoid Arthritis and Inflammatory Bowel Disease

Despite a lack of research regarding its pharmacological activities and chemical constituents, *Urtica atrichocaulis*, a plant indigenous to China, is widely used to treat rheumatoid arthritis. The chemical compositions of the phenolic compound-rich fraction of *U. atrichocaulis* (TFUA) and their anti–rheumatoid arthritis activities were examined. TFUA treatment significantly reduced the effects of adjuvant-induced arthritis and carrageenan-induced paw edema in rats and cotton pellet-induced granuloma and acetic acid-induced writhing reactions in mice [216].

Inflammatory bowel disease (IBD) is a common condition with an unknown cause in Western and Eastern countries. Although recognized therapies exist for the treatment of IBD, their clinical efficacy remains lacking. Plant-derived substances have shown great promise in pharmacological models of inflammation, and a few have been tested in preliminary clinical trials. Isoprenoids, stilbenes, flavonoids, and alkaloids, in addition to structurally related chemicals, are examples of secondary metabolites with anti-inflammatory activities. Most of these chemicals suppress cytokine release by inhibiting NF-κB activity, modulating enzymes and transcription factors [217].

#### 6.14.2. Dietary Polyphenols in the Prevention and Treatment of Allergic Diseases

Body, food, and respiratory allergies comprise the majority of allergy problems. When an insusceptible system becomes sensitive to a typically innocuous allergen, the immune response becomes overwhelmingly oriented to a T-helper type 2 reaction. When the allergen is again introduced, the body releases a critical number of allergy-related intermediaries, resulting in the presentation of symptoms. Our insight into these conditions has improved treatment techniques to balance the acute response or regulate allergen intermediaries, reducing susceptibility to adverse side effects. Polyphenols have been investigated for their antiallergic properties in animal models and human clinical trials. Their mitigating properties have been associated with the recruitment of immune cells to the skin and the anticipation of potential infections following skin breaks. Polyphenols may regulate hypersensitivity through interactions with proteins, and their immediate effects on unfavorable effector cells like pole cells repress the secretion of inflammatory mediators, bringing about sign alleviation. Moreover, the level of cellular damage caused by free radicals during the hypersensitive insult is restricted by polyphenols’ endogenous cancer prevention activities. Generally, polyphenols show promise as antiallergenic agents, potentially influencing various natural pathways and protecting cells during the hypersensitivity reactions, and warrant further consideration [218].

### 6.15. Cardioprotective Activity

According to the literature review, flavonoid and phenolic compounds are found in medicinal plants and shown cardioprotective effects, [219,220,221,222] According to an extensive review by Razavi-Azarkhiavi [223] a cardioprotective function was identified for various phenolic compounds. Although doxorubicin (DOX) is among the most widely used anticancer agents, its clinical application is hampered owing to its cardiotoxicity. Adjuvant therapy with an antioxidant has been suggested as a promising strategy to reduce DOX-induced adverse effects. In this context, many phenolic compounds have been reported to protect against DOX-induced cardiotoxicity [223,224]. *Centaurea transcaucasica* Sosn. Ex Grossh was studied for their protective effects on doxorubicin-treated cardiomyocytes [225]. The cardioprotective effects of phenolic compounds are exerted via multiple mechanisms including inhibition of reactive oxygen species generation, apoptosis, NF-κB, p53, mitochondrial dysfunction, and DNA damage [223].

### 6.16. Effects of Reducing Oxidative Stress in Neurodegenerative Disease

Nanotechnology plays an increasingly important role in reducing oxidative stress, which has been linked to various diseases, such as cancer, AD, and PD [226]; however, the role of this technology in other conditions has yet to be determined. Depending on their antioxidant functionality, nanoparticles can play a critical role in preventing or treating disease by reducing oxidative stress levels, which is a standard function of nano bioactive compounds [227]. The most popular method for delivering antioxidant nanoparticles involves shrinking a natural bioactive molecule to a nanoscale that can rapidly target a site with minimal activity loss [220]. Nanosized bioactive compounds can range in size from 10 to 1000 nanometers, which can enhance both bioactivity and target specificity while simultaneously reducing toxicity and improving safety [228]. The size of the surface activity, carrier toxicity, and the type of bioactive compound are the most important characteristics to determine when designing a nanoparticle for the treatment of PD [229]. Bioactive nanoparticles with smaller sizes can reach a target in the brain target faster than larger nanoparticles. The anti-phagocytosis properties of hydrophilic coatings applied to nano bioactive compounds can prevent their premature degradation, and the nanoparticle carrier should be nontoxic [230]. The process used to prepare nano bioactive compounds determines whether the nano bioactive compounds are packaged in the core or on the surface of the nanoparticles. Curcumin oxidation or hydroxylation can be prevented by packaging curcumin within the core of a nanoparticle [231]. Thiamine-coated nanoparticles have been prearranged on the particle surface to boost antioxidant transport to the brain [232].

The most common neurodegenerative disorder, AD, currently lacks any accepted treatment options. AD is characterized by the loss of cognitive, learning, memory, and language skills. AD is associated with extracellular amyloid-beta plaques and intracellular neurofibrillary tangles containing hyperphosphorylated tau. Approximately 10% of all individuals older than 65 suffer from AD [233]. As a result, current and future patients are likely to face various financial and social issues. Effective interventions for treatment and prevention must be developed rapidly to confront these challenges. Researchers have identified several factors contributing to AD, but the underlying mechanisms that increase neuronal susceptibility to AD with age have not yet been discovered [234]. The study of phenolic compounds in Alzheimer’s disease are shown in Table 6.

As indicated by numerous investigations, the risks of developing certain diseases can be increased or decreased depending on the components of our diets. Diet-associated shifts in trouble have been identified for neurodegenerative diseases [242] and cardiovascular disease. Epidemiological data suggest that particular enhancements (such as malignant growth anticipation agents, vitamins E and B supplements, and polyunsaturated unsaturated fats) [243] and food assortments (such as wine, fish, and vegetables) [244] can prevent or delay cognitive impairments, particularly for AD [244]. Reductions in ROS and cancer prevention and protective agents are critical in neurodegenerative disease. Cellular support, for example, phenolic compounds from dietary plants, might play a role in preventing and treating AD, given the evidence supporting the effects of oxidative stress during AD development. The natural sources of these phenolic compounds and their multitarget reactivity make them potentially valuable tools for managing multifactorial diseases [245]. Another factor that must be considered when examining the effects of dietary phenolic compounds is their capacity to alter the permeability of the blood-brain barrier through impacts on lipid digestion, which might also decrease the risk of stroke [246,247,248,249].

### 6.17. Chemical and Biological Effects of Phenolic Compounds in Cardiovascular Diseases

Increased dietary antioxidant intake has freshly piqued public, media, and science interest in the possibility dietary antioxidants may protect against certain chronic diseases. According to epidemiological evidence, the information of fruits and vegetables may minimize the risks of certain forms of cancer and cardiovascular disease, which is thought to be due to their antioxidant contents. These antioxidants include the well-studied vitamin E and β-carotene, in addition to a wide range of polyphenolic compounds. The presence of flavonoids and phenolic acids in fruit and vegetable-based beverages, such as red wine and green and black teas, has also piqued interest [250].

The oxidized LDL (LDLox) hypothesis suggests LDLox contributes to all stages of atherosclerosis, including inflammatory activation, endothelial degradation, and macrophage enrollment, and the absorption of LDLox by these cells, resulting in the generation of foam cells a hallmark of early atherosclerotic lesions. Although the role of LDLox in atherogenesis is currently well understood, determining the pathways that promote LDLox generation in vivo has proven difficult. Oxidation is thought to occur in the subendothelial space of the arterial wall. A recent study of stable compounds formed via specific pathways has suggested that both ROS and RNS, in addition to other enzymes, such as myeloperoxidase and lipoxygenase, could be involved [145].

Plasma LDL only becomes atherogenic after oxidation. Research suggests that oxidative stress causes atherosclerosis by inducing lipid peroxidation [251]. Extra-virgin olive oil contains polyphenolic compounds that are critical to the prevention of atherosclerotic harm. Inhibitors of 3-hydroxy-3-methylglutaryl-CoA (HMG-CoA) reductase (statins) actively reduce the levels of saturated fatty acids in the plasma (cholesterol) [251]. The effects of polyphenolic composites derived from extra-virgin olive oil on lipid absorption have been the subject of much research [252]. The biological properties of olive oil phenolics are shown in Table 7. 

### 6.18. The Mediterranean Diet and Cardiovascular and Neurodegenerative Diseases

Ancel Keys coined the term Mediterranean diet (MD) during the 1960s to describe the epidemiological observation that Italian and Greek populations had reduced mortality rates and the lower recurrence of harmful neurodegenerative and cardiovascular diseases than other populations [254]. More than 12,000 people from America, Europe, and Asia were included in the Seven Countries Study. Since then, various clinical and epidemiological studies have been published, confirming these discoveries, as demonstrated by the dramatic increase in unique publications regarding the MD since 1999 [255]. The MD involves the high consumption of oats, grains, vegetables, and unsaturated fats (typically from olive oil), the low consumption of red meats, poultry, and submerged unsaturated fats, and the moderate consumption of fish, milk, and dairy items, and moderate ethanol intake (such as drinking wine with meals) [256,257,258]. Some studies of the MD have suggested that adherence to this eating regimen can reduce the risks of developing an assortment of physiological issues, including cardiovascular and cerebrovascular infections, DM, metabolic disorder, malignant growths, and neurodegenerative disease [259]. The Mediterranean dietary pattern includes as distinctive features the moderate intake of red wine and extra virgin olive oil, both of them rich in polyphenolic compounds, such as resveratrol, oleuropein and hydroxytyrosol and their derivatives, which have demonstrated the therapeutic effects [260].

### 6.19. Osteoporosis

Osteoporosis is a bone disease that causes increased skeletal fragility and fractures due to decreased bone mass and microstructural deterioration [173]. The loss of bone mineral density (BMD) is the most common symptom of osteoporosis [261,262]. The most common symptom of osteoporosis is the appearance of fragility bone fractures, which are most commonly found in the vertebrae, wrists, and hip. Such fractures are associated with significant morbidity and death, and despite the availability of various therapies for osteoporosis, the global burden of osteoporotic fractures is growing [263].

Natural medicines offer fewer adverse effects and are better for long-term usage than synthetic drugs. In addition, plant medicines with different chemical ingredients usually have several therapeutic pathways and targets, which is similar to the multiple variables that contribute to osteoporosis pathogenesis [262]. Natural compounds including phytoestrogens with estrogenic effects (e.g., genistein, daidzein, icariin, dioscin, Ginkgo biloba), antioxidant and anti-inflammatory agents (e.g., acteoside, curcumin, resveratrol, *Camellia sinensis*), treatments that exert their effects by multiple actions (e.g., kinsenoside, berberine, *Olea europaea*, *Prunus domestica*, *Allium cepa*) could provide a safer alternative to primary pharmacological strategies for osteoporosis [264].

## 7. Future Aspects

Phenolic compounds commonly found in many plants may represent promising candidates for future medical and pharmaceutical product development. Future studies should consider comparisons of raw plant materials obtained from various geographical areas to determine whether differences exist in the composition of the extracts. Future research should focus on local medicinal plant species and wild or endangered species to explore new phytochemical compounds and expand the number of alternative raw materials for medical and pharmaceutical purposes. The underlying mechanisms associated with some well-known phenols, including the signaling pathways and molecular processes through which they exert their effects, require continued investigation, as this information can be utilized during drug development and targeting. Various medicinal plant cultivars can provide different phenolic compounds with a wide range of biological activities. Therefore, future research should continue to explore different cultivars to identify new compounds [265]. Scientists are constantly searching for medications to combat the coronavirus pandemic (SARS-CoV-2) that has been going on for over a year. Compounds of natural origin, such as phenolic acids and flavonoids, have shown promising antiviral potential in silico study and chosen experimental data. Attachment (disturbance of the interaction between cellular and viral receptors), penetration (inhibition of viral pseudo-particle fusion to the host membrane), replication (inhibition of integrase and 3C-like protease), assembly, and maturation (inhibition of microsomal triglyceride transfer protease) are all stages of the viral life cycle where phenolic compounds inhibit virus multiplication [266]. The black carrot has been shown to help those with type 2 diabetes. This feature is due to the phenolic chemicals found in black carrots. Still, little information regarding the mechanism of action and target enzymes were known phenols and related compounds because they are stable for heating and drying. Moreover, they are unaffected by organic compounds [267]. When used in its pure form, phenol is harmful to tissues. It also has a disagreeable odor.

## 8. Conclusions

Phenolics are a heterogeneous collection of compounds generated as secondary metabolites in plants. Phenolic compounds are aromatic or aliphatic compounds with at least one aromatic ring to which one or more OH groups are connected. Hypertension is a common and sometimes progressive disorder that significantly increases the risk of developing cardiovascular disease and associated complications. Some commonly used ACE inhibitors, such as captopril, benazepril, and enalapril, are associated with side effects, such as the inability to counteract the effects of proteolytic degradation. The inhibition of carbohydrate hydrolyzing enzyme for type 2 DM and cholinesterase inhibition in AD are goals that phenolic compounds can accomplish. Phenolic compounds have anti-inflammatory properties that can be used to treat skin diseases. The most common forms of natural antioxidants are phenols, which feature both antioxidant and anti-inflammatory properties. Despite significant improvements in medicine and pharmacology, researchers still face difficulties understanding the immune system and diseases associated with inflammation. This review described the action and bioavailability of phenolic compounds, including currently known information regarding their biological activities in cancer, rheumatoid arthritis, allergic diseases, cardiovascular disease, and neurogenerative disease. By collecting this information, we can conclude that using phenolic compounds encapsulated in synergistic nanoparticles is likely to enhance our ability to prevent and treat a variety of anti-inflammatory diseases. Through these mechanisms, science can contribute to humanity’s advancement. The disadvantages associated with some phenolic compounds might be mitigated by using a suitable delivery method, such as a nano-based drug delivery system. Several polyphenols that have been examined have performed better when delivered in the core or surface of a nanoparticle than when given as a free soluble compound.

## Figures and Tables

**Table 1:** Mechanisms of anti-inflammatory activity mediated by dietary phenolic compounds [76].

Phenolic Composition	Classification of Phenolic Compounds	Mode of Action	Test Types	References
Genistein Daidzein	Isoflavone Isoflavone	The inhibitor of NF-KB is one of the critical molecular targets of genistein. The inhibitory effect of genistein and Daidzein was moderate (57–72%). Inhibiting STAT-1 activation also was genistein and daidzein expression and NO output	In vitro	[84,93]
Isorhamnetin Pelargonidin	Flavonol Anthocyanin	Isorhamnetin and Pelargonidin both suppressed NF-B activation, but not STAT-1	In vitro	[84]
Kaempferol	Flavonol	The mechanisms through which kaempferol inhibits STAT-1 activation are unknown. However, they may be linked to STAT-1 or its upstream kinase JAK2 phosphorylation	In vitro	[94]
Apigenin	Flavone	Apigenin inhibits the NF-B pathway, which has anti-proliferative, anti-inflammatory, and anti-carcinogenic properties. Apigenin inhibits STAT1-induced CD40 expression, which modulates microglial activation	In vitro	[95,96]
Epicatechin	Flavan-3-ol	The suppression of the NF-B pathway by epicatechin protects against ulcerative colitis. The suppression of transcription factors STAT1 and NF-B in intestinal cells is thought to be the primary cause of this impact	In vitro	[97,98]

**Table 2:** Different polyphenols and their modes of action [135].

Phenolic Compound	Enzyme Inhibition	IC_50_	Source of Abstraction	Study Types	References
Caffeine	AChE	336.8 μmol/L	*Camellia sinensis*	In vitro	[136]
Cinnamic Acid	AChE	8.6 nmol/L	Purified form Acacia honey, *Ocimum africanum*, *Ocimum basilicum*	In vitro	[137]
Resveratrol	AChE, BChE	1.66 μmol/L 1.56 μmol/L	*Vitis amurensis* purified form	In vitro	[138,139,140]
Curcumin	AChE, BChE	58.08 μmol/L	Purified form *Curcuma longa*	In vitro	[141]
Quercetin	AChE, BChE	19.8 μmol/L	*Agrimonia pilosa ledeb*, *Calendula officinalis*, *Gossypium herbaceam* purified form	In vitro	[134]

**Table 3:** Protective effects of plant extracts and phenolic compounds against oxidative stress and inflammation induced by airborne particulate matter [143].

Lessons	Representations	Goals	Ingredients	Reference
In vitro	Keratinocytes from the HaCaT strain	Membrane irritation	Eupafolin from *Phyla nodiflora*	[144]
	Keratinocytes from the HaCaT strain	-	Eupafolin nanoparticles	[145]
	Keratinocytes from the HaCaT strain	Membrane irritation	Nanoparticles comprising 7,3′,4′-trihydroxy isoflavone	[146]
	Fibroblast-like synoviocyte	Membrane irritation	Resveratrol	[147]
	Keratinocytes from the HaCaT strain 3D-skin models	Painful joints	Resveratrol, Resveratryl triacetate	[148]
	EA.hy926 endothelial cubicles, monocytic THP-1 cells	Membrane irritation	Ellagic acid, Punicalagin, Punica granatum abstract	[149]
	Keratinocytes from the epidermis of humans	Irritation	Punicalagin, (−)-Epigallocatechin gallate	[150]
	Dermal fibroblasts from humans	Membrane irritation	(−)-Epigallocatechin gallate	[151]
	Keratinocytes from the HaCaT strain3D-skin models	Membrane irritation	*E. cava* extract, Dieckol	[152]
	Keratinocytes from the HaCaT strain	Membrane irritation	Afzelin from *Thesium chinense*	[153]
	Keratinocytes from the HaCaT strain 3D-skin models	Membrane irritation	Formononetin from *Astragalus mongholicus*	[154]
In vivo	Cockroaches	Cardiac irritation	Chocolate	[155]
	Swine	Bronchial irritation	*Eucheuma cottonii* abstract	[156]
Ex vivo	Keratinocytes from the HaCaT strain Hominoid covering explants	Membrane irritation	*Camellia japonica* abstract	[145]

**Table 4:** Phenolic compounds in psoriasis treatment [173].

Active Constituents	Biological Source	Mechanism of Action	Reference
Quercetin	*Smilax china*	Leucocyte migration and epidermal thickness are reduced	[174]
Capsaicin	*Capsicum annuum*	Because of the release of substance-P, it’s helpful in neurogenic inflammation	[175]
Wrightia dione	*Wrightia tinctoria*	Anti-inflammatory	[175]
Thespesin	*Thespesia populnea*	Retention of the stratum granulosum and significant reduction in the total epidermal thickness	[176]
Chamazulene/matricin	*Matricaria recutita*	By reducing the function of lipoxygenase, has an anti-inflammatory effect	[177]
Silymarin	*Silybum marianum*	It decreases liver damage by inhibiting leukotriene production and cAMP phosphodiesterase action	[178]

**Table 5:** Phenolic compound pharmacological profile against cancer cell lines [215].

Polyphenols	Protective Effects and Mechanisms	Conditions	Study Types
Hydroxytyrosol	Impeding compartment propagation	In hominoid promyelocytic	In vitro
	Tempting caspase-mediated compartment demise via stunning the cubicles in the G0/G1 segment with an affiliated diminution in the compartment proportion in the S and G2/M segments	-	In vitro
Resveratrol	Impeding cubicle spread and downhearted modifiable telomerase bustle	In hominoid colon tumor compartments	In vitro
	Falling the countenance of COX-1, COX-2, c-myc, c-fos, c-jun, converting evolution factor-β-1 and TNF-α	In mouse membrane	In vivo
	Preventing compartment production via intrusive with an estrogen receptor-α-associated PI3K lane	In estrogen-responsive MCF-7 human breast cancer compartments	In vitro
	Impeding nitrobenzene (NB)-DNA adducts	In male Kunming mice adducts	In vivo
Chlorogenic acid	Preventing the development of DNA single strand interruptions	In supercoiled pBR322 DNA	In vitro
Quercetin Luteolin	Stalling EGFR tyrosine kinase movement	In MiaPaCa-2 cancer cubicles	In vitro
EGCG	Obstructing telomerase	In human cancer compartments	In vitro
Silymarin Hesperetin Quercetin Daidzein	Relating with *p*-glycoprotein and modulating the activity of ATP-binding cassette truck, breast cancer struggle protein (BCRP/ABCG2)	In two separate BCRP-overexpressing cell lines	In vitro
Myricetin Apigenin Quercetin Kaempferol	Hindering human CYP1A1 activities Impeding the construction of diolepoxide 2(DE2) and B[a]P beginning	On 7-ethoxyresorufin *o*-deethylation	In vitro

**Table 6:** Some proposed mechanisms for the beneficial effects of polyphenols in Alzheimer’s disease.

Polyphenols	Proposed Mechanism of Action	Study Type	Reference
Resveratrol	Encourages deprivation of Ab via proteasome	In vitro	[235]
	Protects against Ab-mediated cell death via PKC phosphorylation	In vitro	[236]
EGCG	Hinders creation, delay and steadiness of Ab fibrils in vitro	In vitro	[237]
	Keeps since Ab-induced apoptosis	In vitro	[238]
	Encourages non-amyloidogenic way in animal and cell models	In vitro	[239]
Curcumin	Hinders construction of Ab fibrils in vitro	In vitro	[239]
	Diminishes oxidative stress and plaques construction in APPSw transgenic mice	In vitro	[240]
	Shields cells since oxidative Ab insult	In vitro	[241]

**Table 7:** Biological properties of olive oil phenolics [253].

Polyphenolic Composite	Process of Accomplishment	Study Types	Beneficial Result on Anthropoid Wellbeing
Hydroxytyrosol, protocatechuic acid, phenyl ethanol-elenolic acid, caffeic acid and are some of the compounds checked in oleuropein.	The embarrassment of HMG-CoA reductase, Low-density lipoprotein oxidation in vitro and in vivo shyness of thromboxane B_2_ and, as a result, thrombocyte accumulation	In vitro	Stoppage of cardiovascular sicknesses
Lignans and Secoiridoids	Repressive act on the action of diminution of superoxide formation xanthine oxidase and lignans performance as anti-estrogens and improvement sex hormone obligatory globulin	In vitro	Stoppage of tumoral sicknesses
Hydroxytyrosol and other polyphenolics	Repressing achievement on lipo-oxygenase and cyclo-oxygenase diminish inflammatory molecule formation such as leukotriene B and thromboxane B2	In vitro	Anti-inflammatory motion
Oleuropein; verbascoside (hydroxytyrosol and tyrosol)	The shyness of viral and bacterial evolution and motion	In vitro	Antimicrobial and antiviral motion

## Data Availability

Available data are presented in the manuscript.
